# Programme‐Led and Focused Interventions for Recent Onset Binge/Purge Eating Disorders: Use and Outcomes in the First Episode Rapid Early Intervention for Eating Disorders (FREED) Network

**DOI:** 10.1002/eat.24343

**Published:** 2024-11-30

**Authors:** Karina L. Allen, Laura Courtney, Philippa Croft, Lucy Hyam, Regan Mills, Katie Richards, Muhammad Ahmed, Ulrike Schmidt

**Affiliations:** ^1^ Eating Disorders Service, Maudsley Hospital, South London and Maudsley NHS Foundation Trust London UK; ^2^ Centre for Research in Eating and Weight Disorders, Institute of Psychiatry, Psychology and Neuroscience, King's College London London UK; ^3^ Department of Clinical Education and Health Psychology, University College London London UK; ^4^ Centre of Implementation Science, Institute of Psychiatry, Psychology and Neuroscience, King's College London London UK

**Keywords:** binge eating disorder, bulimia nervosa, cognitive behavioral therapy, early intervention, eating disorders, focused interventions, guided self‐help, low‐intensity interventions

## Abstract

**Objective:**

We aimed to compare use of, and outcomes from, programme‐led and focused interventions (guided self‐help and 10 session cognitive behavioral therapy for eating disorders [CBT‐T]) relative to other psychological therapies (including group and individual CBT for eating disorders [CBT‐ED]) in a national sample of emerging adults receiving early intervention for a non‐underweight binge/purge eating disorder.

**Method:**

Data were drawn from 54 English eating disorder services using the First Episode Rapid Early Intervention for Eating Disorders (FREED) model. Participants (*N* = 1097) had a mean age of 18.95 years (SD 2.42) and diagnoses of bulimia nervosa (*n* = 506; 45%), binge eating disorder (*n* = 121; 11%), another specified feeding or eating disorder (*n* = 460; 42%), or an eating disorder, unspecified (*n* = 10, 1%). Linear mixed models were used to assess for effects of time and treatment on binge eating and purging, eating disorder psychopathology, depression/anxiety, and body mass index.

**Results:**

11% (*n* = 117) of patients received guided self‐help and 24% (*n* = 268) received CBT‐T. Baseline eating disorder psychopathology and depressive/anxiety symptoms did not differ significantly across the guided self‐help, CBT‐T, group CBT‐ED, and individual CBT‐ED conditions. All treatments were associated with significant reductions in symptoms over time. GSH and CBT‐T performed comparably to longer CBT‐ED.

**Discussion:**

We provide additional evidence for the effectiveness of GSH and CBT‐T in the treatment of non‐underweight binge/purge eating disorders. Programme‐led and focused interventions may be under‐utilized and future research should explore when they are offered, and when not, both within and outside of early intervention settings.

**Summary**

- Guided self‐help (GSH) and 10‐session cognitive behavioral therapy for eating disorders (CBT‐T) are effective treatments for binge/purge eating disorders.
- Despite being effective, these treatments were only offered to 35% of patients in this national sample.
- Only 11% were offered GSH, which is the recommended first‐line treatment for BN and BED. When GSH and CBT‐T were offered, they resulted in similar clinical improvements as longer therapies.
- Improving use of programme‐led and focused treatments like GSH and CBT‐T may help more people to access timely, effective eating disorder treatment.

## 1 Introduction

Eating disorders (EDs) are distressing and disabling mental health disorders which affect up to 15% of cisgender women and 5% of cisgender men (Hay et al. 2023; Micali et al. 2017). Despite their impact and prevalence, access to treatment is often delayed or does not occur at all (Hart et al. 2011; Weissman and Rosselli 2017). A systematic review of Duration of Untreated ED (DUED) found that, when treatment does occur, mean DUED is 30 months for anorexia nervosa (AN), 53 months for bulimia nervosa (BN), and 67 months for binge eating disorder (BED) (Austin et al. 2021). This review captured literature from before the COVID‐19 pandemic, and demand on ED services is known to have increased since (e.g., Viljoen et al. 2023). Improving access to treatment and reducing DUED requires promotion of help‐seeking but also sufficient resources to meet treatment demand (Kazdin, Fitzsimmons‐Craft, and Wilfley 2017). Currently, ED services are under‐resourced to meet demand (Viljoen et al. 2023), and there is not an adequate workforce available to provide ED‐focused psychological therapies via the ‘dominant model of treatment delivery’ (Kazdin, Fitzsimmons‐Craft, and Wilfley 2017). This refers to the historical tendency for psychological therapies to be delivered via individual, in‐person sessions with a highly trained mental health professional in a specialist mental health or ED treatment center. This approach is resource‐intensive and limits the accessibility and availability of treatment. Novel models of intervention delivery have been proposed (e.g., Kazdin 2024), with key areas of emphasis including task‐shifting (where possible) to a less highly trained workforce and making use of “disruptive innovations” which are more readily scalable, including digital technologies.

Programme‐led and focused interventions offer opportunities for disrupting the dominant model of treatment delivery and reducing the gap between resources and demand. Programme‐led interventions have been defined as interventions where the expertise is in the programme (e.g., within a book or digital programme) rather than the therapist/professional, and focused interventions as those which require 50% or less therapist time than the standard alternative treatment (Davey et al. 2023). Evidence‐based programme‐led interventions for EDs include guided self‐help (GSH) based on cognitive behavioral therapy (CBT) for bulimia nervosa (BN) and binge eating disorder (BED). Focused treatment is available in 10‐session CBT for non‐underweight EDs (CBT‐T) (Waller et al. 2019). These interventions may be offered as standalone treatments or as part of a stepped care pathway, where initial non‐response to GSH or CBT‐T sees patients “stepped up” to a more senior clinician and/or a longer form of therapy.

GSH based on CBT is recommended by the UK National Institute for Health and Care Excellence (NICE) as the first‐line treatment for BN and BED (National Institute for Health and Care Excellence 2017). GSH is also recommended in other international clinical guidelines for BN and BED, although without specific advice about when to offer this relative to other treatments (Hilbert, Hoek, and Schmidt 2017). There is good evidence for the effectiveness of GSH in reducing binge eating and ED psychopathology, often with outcomes that are comparable to those from longer forms of CBT for EDs (CBT‐ED, usually 20 sessions) (Traviss‐Turner, West, and Hill 2017; Wilson and Zandberg 2012). The addition of a “guide” or “coach” improves outcomes compared to pure self‐help, and support is generally provided via 4 to 9 sessions, each lasting 20–30 min, over 12 to 16 weeks. GSH is cost‐effective as well as clinically effective (König et al. 2018; Lynch et al. 2010) and allows for task‐shifting from highly trained psychological therapists to more junior “coaches” who can provide guided support using a structured protocol and under expert supervision (Davey et al. 2023).

Since 2019, CBT‐T has provided another CBT‐based treatment that is quicker than, but comparable in effectiveness to, 20‐session CBT‐ED (Waller et al. 2019). A systematic review and meta‐analysis of 10 studies found that 61% of those who start CBT‐T complete it, and 65% of treatment completers achieve a good outcome (Keegan, Waller, and Wade 2022). These results were from studies of patients with a pre‐treatment Body Mass Index (BMI) > 18.5, and so included those with BN and BED but also other forms of other specified feeding or eating disorders (OSFED). Like GSH, CBT‐T may be delivered by relatively junior therapists and provides opportunities for task shifting as well as cost savings, improved treatment throughput, and a more diverse workforce that is better able to meet the needs of a heterogeneous patient population (Davey et al. 2023).

There has been one study directly comparing GSH to CBT‐T (Wade, Ghan, and Waller 2021). This employed a randomized controlled design to allocate 98 adolescents and adults (≥ 15 years) to 10 sessions of CBT‐T (*n* = 46) or 10 sessions of GSH CBT with an emphasis on motivation (*n* = 52). Both treatments were associated with statistically and clinically significant reductions in global ED psychopathology, clinical impairment, and symptoms of depression, anxiety, and stress. There were no overall group differences, but moderator analyses found that patients with low motivation to change at pre‐treatment did better with GSH than with CBT‐T. Of note, however, this study offered 10 sessions in the GSH condition, which is more than many forms of GSH provide.

Group psychotherapy for EDs, including group CBT‐ED, also produces treatment effects that are comparable to those from longer individual therapies (e.g., Grenon et al. 2017). In current UK treatment guidelines, group CBT‐ED is recommended as the second ‘step’ in treatment for BED if GSH is unacceptable, contraindicated, or ineffective after 4 weeks (National Institute for Health and Care Excellence 2017). In contrast, individual CBT‐ED is recommended for BN if GSH is not possible or effective.

To date, no studies have directly compared GSH, CBT‐T, group CBT‐ED, and individual CBT‐ED. Evaluating how these different treatments are used, and how they compare to each other, will help improve our understanding of their relative effectiveness and may inform treatment matching. This would have benefits for resource allocation and improving access to effective ED care. While CBT‐based interventions are the leading evidence‐based treatment option for binge/purge EDs, there is also evidence for the effectiveness of interpersonal psychotherapy (Hilbert, Hoek, and Schmidt 2017; Miniati et al. 2018) and family‐based interventions for adolescents and emerging adults (Schmidt et al. 2007). In this context, the current study aimed to compare use of, and outcomes from, programme‐led and focused interventions (GSH and CBT‐T) relative to group and individual CBT‐ED and other psychological therapies in a naturalistic, national sample of emerging adults receiving early intervention for a recent‐onset, non‐underweight binge/purge ED. Data were drawn from 54 services across England using the evidence‐based First Episode Rapid Early Intervention for Eating Disorders (FREED) model.

## 2 Method

### 2.1 Design

The FREED Network includes all adult or all‐age English National Health Service (NHS) ED services that offer a FREED early intervention pathway. FREED consists of a service model and a care package and was developed to provide evidence‐based early intervention for 16‐ to 25‐year‐olds with an ED of less than 3 years duration (Schmidt et al. 2016). The service model focuses on proactive engagement and provides waiting time targets for assessment and treatment, with a central focus on reducing DUED. The care package involves adapting existing evidence‐based treatments for EDs (including GSH, CBT‐T, and CBT‐ED) to the developmental needs of emerging adults with a recent‐onset ED. This includes an even greater emphasis on early behavioral change, as well as attention to emerging adulthood, transitions (e.g., out of education, into work, in living circumstances), social media/app use, and family involvement (Allen et al. 2020). Between 2018 and 2023, FREED scaled from 8 to 54 NHS Trusts. FREED Network services provide core, de‐identified FREED patient data on a quarterly basis. These data are managed by the FREED National Team at King's College London (KCL) and South London and Maudsley NHS Foundation Trust (SLaM), overseen by the SLaM Information Governance Team and with an approved Data Protection Impact Assessment. Patients can opt out of data sharing. Additional information on FREED can be found at www.FREEDfromED.co.uk.

This study focused on patients who were referred to a FREED Network service between September 2018 (when FREED started national scaling) and December 2023; who were FREED eligible at assessment (aged 16–25 years with DUED < 3 years) and had started treatment by December 2023; who were not significantly underweight (BMI > 18.5); and who may have been suitable for GSH or CBT‐T. This included patients diagnosed with BN, sub‐threshold/atypical BN, BED, sub‐threshold/atypical BED, purging disorder, another OSFED, or “ED, unspecified.” Participants with a stated diagnosis of anorexia nervosa (AN), atypical AN, or avoidant restrictive food intake disorder (ARFID) were not included.

### 2.2 Participants

In total, 8100 patients were referred to FREED Network services between September 2018 and December 2023 and 1097 (14%) met the above criteria for inclusion in this study. Reasons for exclusion were: *n* = 1539 referred patients (19%) were still awaiting assessment; *n* = 2729 assessed patients met criteria for a non‐underweight binge/purge ED but were waiting to start treatment (*n* = 2341, 28.9%), disengaged after assessment (*n* = 331, 4.1%), or moved out of area (*n* = 57, 0.7%); and *n* = 2735 (33.8%) assessed patients had a diagnosis of AN, atypical AN or ARFID.

The 1097 participants had a mean age of 18.95 years (SD 2.42, range 16–25) with a mean DUED of 17.47 months (i.e., 1.45 years) (SD 9.60 months, range 0–36). Diagnoses were: 46% with BN (*n* = 506), 11% with BED (*n* = 121), 42% with OSFED (*n* = 460), and 1% (*n* = 10) with an ED unspecified. Within the OSFED group, 37 patients were specifically diagnosed with atypical/sub‐threshold BN and 11 were specifically diagnosed with purging disorder. An OSFED sub‐category was not specified for the remainder. It is possible that some participants with atypical AN were within this group. Mean baseline BMI was 24.40 (SD 6.55, range 18.51–60.9).

### 2.3 Measures

#### 2.3.1 Demographic, diagnostic, and treatment information

Services reported patients' age, DUED, ED diagnosis, BMI, waiting times for assessment and treatment, the type and amount of treatment provided, and whether treatment was completed. Training was provided in how to calculate DUED (months from onset of a clinically significant ED to the FREED assessment). Diagnosis could be provided to DSM‐5 or ICD‐10 nomenclature, with categories summarized to DSM‐5 criteria for this study. Under the data sharing agreement, personally identifying or “special category” data could not be shared. This included information on gender, race, and ethnicity.

#### 2.3.2 Eating Disorder Examination‐Questionnaire (EDE‐Q; Fairburn and Beglin 2008)

The EDE‐Q is a well‐validated self‐report measure of ED cognitions and behaviors over the past 28 days. This study made use of the Global score as well as behavioral items to determine the presence and frequency of objective binge eating and purging (self‐induced vomiting + laxative misuse). The Global score is calculated by taking the mean of Restraint, Eating Concern, Weight Concern and Shape Concern subscales and assesses overall ED psychopathology. Scores can range from 0 to 6 with a score ≥ 2.8 suggesting clinically concerning ED symptoms in women (Mond et al. 2006).

#### 2.3.3 Clinical Outcomes in Routine Evaluation‐10 (CORE‐10; Barkham et al. 2013)

The CORE‐10 assesses anxiety and depressive symptoms over the past 2 weeks. It creates a Total score ranging from 0 to 3, where higher scores reflect greater distress. The CORE‐10 was originally developed for use in UK primary mental health care settings and is widely used across UK mental health services. It shows good internal consistency and validity (Barkham et al. 2013).

#### 2.3.4 Procedure

Each FREED Network site had a FREED Champion responsible for compiling de‐identified data for sharing on a quarterly basis. Data were manually sent to the FREED National team for central compiling and summarizing. Analyses for this research used data shared up to the end of December 2023. Services differed in their processes for collecting questionnaire data and completion rates varied within and across services with high rates of missing data overall (see below). Ideally, patients were asked to complete the EDE‐Q and CORE‐10 at assessment, pre‐treatment, mid‐treatment, and post‐treatment. Assessment and pre‐treatment data points were combined for this research as data were often only available at one of the timepoints. Where both timepoints were available, the assessment measure was retained as the earlier data point.

### 2.4 Statistical Analyses

All analyses were conducted in SPSS 27.

#### 2.4.1 Treatment type and use

The types of treatments offered to patients were reported descriptively. One‐way Analysis of Variance (ANOVA) was used to compare participant demographic details (age, DUED) and waiting times across the treatment groups, as well as the mean number of sessions attended. Tamhane's T2 was used for the post hoc group comparisons due to unequal variance. Chi‐square was used to compare treatment completion rates across groups.

#### 2.4.2 Treatment outcomes

Linear mixed models with maximum likelihood estimation were used to assess for effects of time (pre‐, mid‐, post‐treatment) and treatment group (GSH, CBT‐T, CBT‐ED, other psychological therapies) on Global EDE‐Q scores, CORE‐10 scores, and BMI. Generalized linear mixed models using negative binomial regression were used to assess for effects of time and treatment group on binge eating and purging episodes/month. Negative binomial regression is suited to count variables (i.e., binge/purge episodes) that are over‐dispersed due to non‐normal distribution and a skew towards zero (i.e., as patients stop binge eating/purging).

Time and treatment group were initially entered as fixed effects before re‐running models with an interaction effect between time and treatment group. Fixed effects test for overall differences over time and across treatment groups, whereas interaction effects test whether any changes over time differ between the treatment groups (i.e., whether one group showed greater improvements than another). Random effects were included to account for repeated measurement and the clustering of patients within services.

Models were run without adjusting for baseline variables, and then with baseline age, DUED, and waiting time for treatment (days between referral and treatment) added as covariates. A second set of analyses were run to add number of treatment sessions as a further covariate, to account for treatment dose/completion. Finally, for Global EDE‐Q scores, CORE‐10 scores, binge eating episodes, and purging episodes, additional models were run adding baseline BMI as a covariate.

## 3 Results

### 3.1 Missing Data

All participants had information provided regarding ED diagnosis and the type of treatment they received. Missing data for other baseline variables were: 37% for Global EDE‐Q scores, 35% for binge eating and purging scores, and 61% for CORE‐10 depression/anxiety scores. At post‐treatment, missing data were observed in 80%–81% of participants for BMI, Global EDE‐Q scores, binge eating and purging; and for 90% of participants for CORE‐10 depression/anxiety scores. This meant that outcomes to post‐treatment were available for *n* = 208–221 for BMI and ED variables, and *n* = 113 for CORE‐10 scores. Complete data across all values were available in 29% of cases. From discussions with FREED Network services, reasons for missing data include patients not wanting to complete questionnaire measures, clinicians forgetting or not prioritizing the collection of data, and data not being entered into FREED trackers for sharing. Missing data at post‐treatment were also significantly more likely when patients discontinued treatment early (*p*s < 0.001 for all post‐treatment variables) compared to treatment completers. Missing data ranged from 78% to 86% across post‐treatment variables for treatment non‐completers, and from 51% to 73% for treatment completers.

Little's MCAR tests were significant, confirming that data were not missing completely at random (*χ* ^*2*^ = 2127, *df* 1887 *p* < 0.001). Given the amount and non‐random nature of missing data, multiple imputation was not possible, but linear mixed models are able to make full use of available data. All analyses were run as intent‐to‐treat and as noted, we adjusted for number of treatment sessions to help account for treatment dose and non‐completion.

### 3.2 Treatment Type and Use

Over three‐quarters of patients (*n* = 847/77%) received a CBT‐based intervention: GSH, CBT‐T, group CBT‐ED, or individual CBT‐ED (see Table 1). A further 3% received brief support broadly under the umbrella of CBT, in the form of psychoeducation or motivational support. Other interventions are summarized in Table 1. For GSH, not all services reported on the type of GSH used but where information was provided this included use of the books *Overcoming Binge Eating* (Fairburn 2013) and *Getting Better Bite by Bite* (Schmidt, Treasure, and Alexander 2015) and the online programme *Overcoming Bulimia Online* (https://overcomingbulimia.com/obo‐course/). Support was provided via emails, telephone calls, virtual sessions, and brief in‐person sessions.

**TABLE 1:** Treatments received by non‐underweight patients in the FREED Network (*N* = 1097), along with mean age and duration of untreated eating disorder (DUED, in months) for each treatment group.

	*n*	%	Age (M[SD])	DUED (M[SD])
Psychoeducation only	27	2.5%	19.13 (2.20)	15.96 (9.64)
Motivational support only	10	0.9%	19.10 (1.29)	16.70 (6.91)
Guided self‐help (GSH)	117	10.7%	19.57 (2.55)	18.48 (9.95)
Cognitive Behavior Therapy‐10 (CBT‐T)	268	24.4%	19.40 (2.58)	18.24 (9.85)
Group‐based Cognitive Behavior Therapy for Eating Disorders (CBT‐ED)^a^	105	9.6%	18.70 (2.41)	17.61 (9.61)
Individual CBT‐ED	357	32.4%	18.75 (2.35)	17.11 (9.54)
Family‐based treatment	5	0.5%	16.71 (0.49)	11.86 (6.23)
Maudsley Anorexia Nervosa Treatment for Adults (MANTRA)	29	2.6%	18.93 (2.62)	14.72 (8.42)
Specific Supportive Clinical Management for Anorexia Nervosa (SSCM)	43	3.9%	17.90 (1.52)	19.10 (9.93)
Other individual therapy^b^	136	12.4%	18.90 (2.42)	17.11 (9.15)

Abbreviation: DUED = duration of untreated eating disorder.

^a^ Including *n* = 4 group GSH, *n* = 8 group CBT‐T, *n* = 86 group CBT‐ED.

^b^ Including *n* = 12 cognitive analytical therapy, *n* = 3 compassion focused therapy, *n* = 5 DBT, *n* = 7 interpersonal psychotherapy, *n* = 119 other or unspecified individual psychological therapy.

There were 72 participants who received either MANTRA (*n* = 29) or SSCM (*n* = 43). These are evidence‐based treatments for AN and their use in this sample of non‐underweight patients may reflect provision to patients with a history of AN who had weight restored prior to this course of outpatient therapy, or with atypical AN within the OSFED diagnostic category. There were also 5 participants who received family‐based treatment (FBT) for their ED. The remaining 136 participants received another individual psychological therapy.

Mean DUED did not differ significantly across the different treatment conditions, *F*(9, 121) = 1.32, *p* = 0.222 (see Table 1), but there was a significant difference in baseline age, *F*(9, 24) = 4.08, *p* < 0.001. This stemmed from participants who received FBT being significantly younger, on average, than those in all other treatment groups (*p*s = 0.011‐ < 0.001); and participants who received SSCM being significantly younger than those who received GSH or CBT‐T (*p*s < 0.001) (see Table 1). Given the small group sizes for FBT and SSCM, these results should be seen as provisional.

Subsequent analyses excluded the participants who received MANTRA and SSCM as well as the 5 individuals who received family‐based treatment and the 37 who received only psychoeducation or motivational support. This reflected small group sizes in these conditions as well as the likelihood of these participants being meaningfully different (either due to an AN‐like presentation or their suitability for family‐based work or very brief support) compared to other participants. As a result, the effective sample size for treatment comparison analyses was *N* = 983.

There were significant between‐group differences in waiting times to assessment, *F*(4, 928) = 7.62, *p* < 0.001, and treatment, *F*(4, 932) = 9.52, *p* < 0.001, due to patients who received an ‘other’ individual therapy waiting significantly longer for both assessment and treatment than other patients. For assessment, the “other” group waited an average of 36.89 days (SD 44.43) compared to 16.92–21.44 days across the CBT‐based groups (SD 18.72–33.16). For treatment, the “other” group waited an average of 121.40 days (SD 102.60) compared to 61.20–78.01 days across the CBT‐based groups (SD 49.37–73.47). There was also a significant between‐group difference in wait between assessment and treatment, *F*(4, 928) = 5.40, *p* < 0.001, which again stemmed from patients who received an “other” individual therapy waiting significantly longer between assessment and treatment (*M* = 84.50 days, SD 78.18) than those who received GSH (*M* = 44.27 days, SD 36.03), CBT‐T (*M* = 56.81 days, SD 63.51), and individual CBT‐ED (*M* = 57.98 days, SD 66.32) (*p*s = 0.027‐ < 0.001). Patients who received individual CBT‐ED also waited significantly longer between assessment and treatment than those who received GSH (*p* = 0.048).

Overall, 17% of patients discontinued treatment early and this did not differ significantly across the treatment groups, *χ* ^2^(4) = 6.05, *p* = 0.196. Rates by group were: 18% for GSH, 16% for CBT‐T, 10% for group CBT‐ED, 23% for individual CBT‐ED, and 7% for another individual therapy.

The mean number of attended treatment sessions did differ significantly by group, *F*(4,356) = 5.90, *p* < 0.001. As might be expected, GSH patients attended significantly fewer sessions (*M* = 5.73, SD = 2.93, range 1–16) than CBT‐T (*M* = 8.11, SD = 5.23, range 1–20), group CBT‐ED (*M* = 8.13, SD = 8.48, range 1–26), individual CBT‐ED (M = 10.90, SD = 8.48, range 1–53), or “other” individual therapy (*M* = 11.85, SD = 8.15, range 1–34) patients. Additionally, CBT‐T patients attended significantly fewer sessions than those who received individual CBT‐ED.

### 3.3 Treatment Outcomes

**FIGURE 1:**
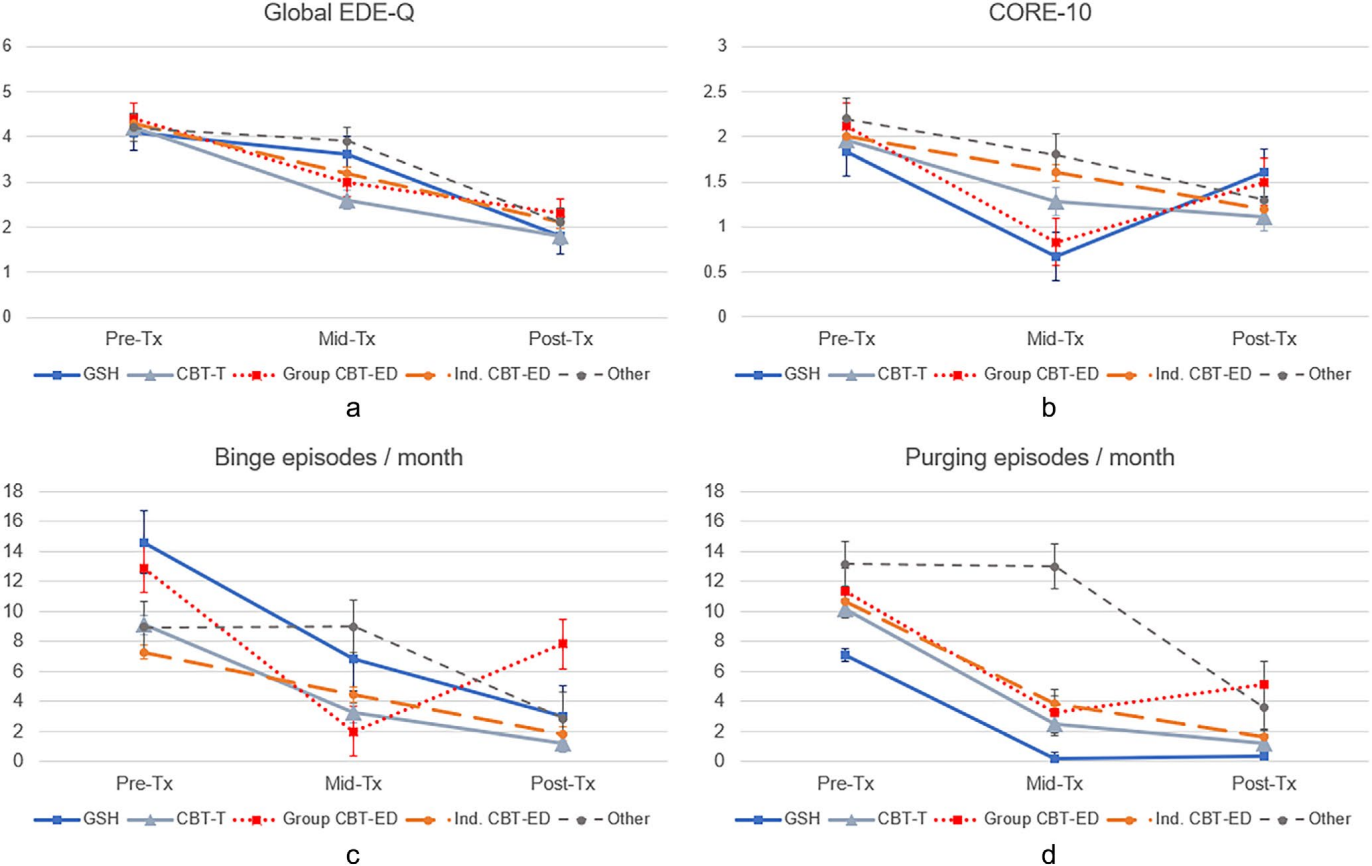
Mean Global Eating Disorder Examination‐Questionnaire (EDE‐Q), CORE‐10 depression/anxiety, binge eating/month and purging/month, by time and treatment condition.

**FIGURE 2:**
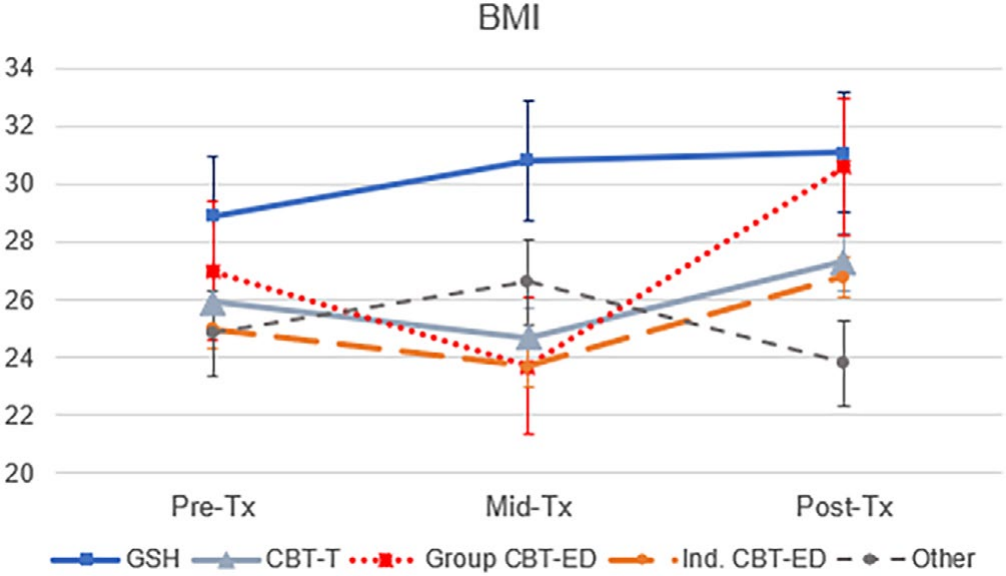
Mean Body Mass Index (BMI) by time and treatment condition.

Changes over time in mean Global EDE‐Q scores, CORE‐10 scores, binge eating episodes, and purging episodes are shown in Figure 1, by treatment group. Changes in mean BMI are shown in Figure 2. Estimated means and group sizes are also provided in Table 2 and full linear mixed model results are provided in Table S1.

**TABLE 2:** Estimated marginal means (and standard errors) by treatment group and time, from unadjusted linear mixed models.

	Pre‐treatment	Mid‐treatment	Post‐treatment	Effects
Global EDE‐Q (*n* = 592)				
GSH	4.14 (0.14)	3.60 (0.64)	1.77 (0.44)	Time *p* < 0.001* Group *p = 0*.495 Time × group *p* = 0.542
CBT‐T	4.22 (0.09)	2.62 (0.36)	1.82 (0.19)	
Group CBT‐ED	4.37 (0.15)	3.00 (0.45)	2.30 (0.38)	
Individual CBT‐ED	4.30 (0.07)	3.19 (0.19)	2.13 (0.16)	
Other individual	4.18 (0.14)	3.91 (0.45)	2.11 (0.34)	
CORE‐10 (*n* = 392)				
GSH	1.83 (0.10)	0.67 (0.39)	1.57 (0.33)	Time *p* < 0.001* Group *p = 0*.049* Time × group *p* = 0.213
CBT‐T	1.96 (0.08)	1.28 (0.22)	1.12 (0.15)	
Group CBT‐ED	2.11 (0.11)	0.83 (0.39)	1.54 (0.28)	
Individual CBT‐ED	2.04 (0.05)	1.58 (0.11)	1.21 (0.11)	
Other individual	2.20 (0.13)	1.84 (0.22)	1.32 (0.33)	
Binge eating (*n* = 619)				
GSH	14.59 (1.90)	6.80 (3.26)	3.00 (1.10)	Time *p* < 0.001* Group *p* < *0*.001* Time × group *p* < 0.001*
CBT‐T	9.10 (0.78)	3.24 (0.90)	1.24 (0.22)	
Group CBT‐ED	12.89 (1.84)	2.00 (0.82)	7.85 (2.31)	
Individual CBT‐ED	7.28 (0.48)	4.46 (0.71)	1.86 (0.25)	
Other individual	8.94 (1.20)	9.00 (3.35)	2.86 (0.72)	
Purging (*n* = 617)				
GSH	7.12 (0.95)	0.20 (0.10)	0.40 (0.24)	Time *p* < 0.001* Group *p* < *0*.001* Time × group *p* < 0.001*
CBT‐T	10.18 (0.87)	2.50 (0.79)	1.22 (0.22)	
Group CBT‐ED	11.37 (1.66)	3.25 (1.32)	5.15 (1.56)	
Individual CBT‐ED	10.64 (0.69)	3.83 (0.63)	1.67 (0.23)	
Other individual	13.14 (1.73)	13.00 (2.10)	3.57 (0.88)	
BMI (*n* = 690)				
GSH	28.87 (0.81)	30.80 (2.44)	31.15 (2.89)	Time *p* = 0.278 Group *p* < *0*.001* Time x group *p* = 0.558
CBT‐T	25.93 (0.51)	24.71 (1.28)	27.31 (1.14)	
Group CBT‐ED	27.06 (0.81)	23.74 (2.99)	30.60 (3.27)	
Individual CBT‐ED	24.99 (0.39)	23.67 (0.74)	26.78 (0.96)	
Other individual	24.85 (0.80)	26.58 (1.73)	23.80 (1.89)	

Abbreviations: BMI = body mass index; CORE‐10 = clinical outcomes in routine evaluation‐10; GSH = guided self‐help; CBT‐T = 10‐session cognitive behavioral therapy (CBT) for non‐underweight eating disorders; CBT‐ED = CBT for eating disorders; EDE‐Q = eating disorder examination‐questionnaire.

For Global EDE‐Q scores, there was a statistically significant effect of time, *F*(2,256) = 203, *p* < 0.001, such that mean Global EDE‐Q scores decreased over time for all treatment groups (see Figure 1a). There was not a significant overall effect of treatment group, *F*(4,762) = 0.85, *p* = 0.495, nor was there a significant interaction between time and treatment group, *F*(8,238) = 0.87, *p* = 0.542. This pattern of results did not change after adjusting for baseline age, DUED, or waiting time for treatment; or when adding number of attended treatment sessions; or when adding baseline BMI. None of these covariates significantly predicted EDE‐Q scores (see Table S1).

For CORE‐10 scores, there was a statistically significant effect of time, *F*(2,174) = 47.90, *p* < 0.001, and of treatment group, *F*(4,501) = 2.40, *p* = 0.049, but no significant interaction between time and group, *F*(8,163) = 1.37, *p* = 0.213. As shown in Figure 1b, patients who received an “other” psychological therapy had higher CORE‐10 scores, on average, than the GSH and CBT‐T groups. After adjusting for covariates, the difference between the other psychological therapy group and the GSH group remained statistically significant, but the difference with CBT‐T did not (see Table S1). Of the added covariates, only age was significant in the model, reflecting a small negative association between younger age and higher CORE‐10 scores (estimate = −0.04, *p* = 0.020) (Table S1).

For binge eating, there was a statistically significant effect of time, Wald *χ* ^2^ (2) = 205.08, *p* < 0.001, of treatment group, Wald *χ* ^2^ (4) = 45.08, *p* < 0.001, and in the interaction between time and treatment group, Wald *χ* ^2^ (8) = 31.97, *p* < 0.001. As shown in Figure 1c, patients who received GSH and group CBT‐ED reported significantly more binge eating at baseline than patients who received CBT‐T, individual CBT‐ED, or another psychological therapy. Patients who received another psychological therapy also reported significantly more binge eating than patients who received individual CBT‐ED. The group CBT‐ED condition showed a marked decrease in binge eating from pre‐ to mid‐treatment, with an increase in mean binge eating from mid‐ to post‐treatment. Patients in the GSH, CBT‐T, and individual CBT‐ED conditions showed decreases between each time point, while those who received another psychological therapy showed reductions from mid‐ to post‐treatment only. The pattern of results was similar when adjusting for baseline age, DUED, and waiting time for treatment; when adding number of treatment sessions; and when adding BMI. However, in the adjusted models, binge eating was no longer significantly elevated in the GSH and group CBT‐ED groups compared to other treatment conditions. In the final adjusted model, DUED (estimate = 0.13, *p* = 0.031), waiting time for treatment (estimate = 0.01, *p* = 0.031) and BMI (estimate = 0.04, *p* < 0.001) significantly predicted binge eating alongside time and treatment group, reflecting small positive associations between these covariates and greater binge eating (see Table S1).

For purging, there was a statistically significant effect of time, Wald *χ* ^2^ (2) = 324.05, *p* < 0.001, of treatment group, Wald *χ* ^2^ (4) = 36.81, *p* < 0.001, and in the interaction between time and treatment group, Wald *χ* ^2^ (7) = 24.80, *p* < 0.001. As shown in Figure 1d, patients who received another psychological therapy reported higher levels of purging than those in GSH, CBT‐T, and individual CBT‐ED. Patients who received group CBT‐ED also reported higher levels of purging, on average, than those who received GSH. As with binge eating, purging reduced significantly between pre‐ and mid‐treatment for patients who received GSH, CBT‐T, group CBT‐ED, and individual CBT‐ED, but not for patients who received another psychological therapy. Patients who received group CBT‐ED then showed a slight increase in purging between mid‐ and post‐treatment, while other patients experienced stable or reducing purging over this time period (see Figure 1d). This pattern of results remained after adjusting for baseline age, DUED, and waiting time, when adding number of treatment sessions, and when adding baseline BMI. Of these covariates, only age and BMI were significant predictors of purging, with a small negative association between younger age and greater purging (estimate = −0.10‐, *p < 0*.001) and between lower BMI and greater purging (estimate = −0.04, *p* < 0.001) (Table S1).

For BMI, there was no statistically significant effect of time, *F*(2,231) = 1.35, *p* = 0.272, but there was a significant effect of treatment group, *F*(4,906) = 7.91, *p* < 0.001. The interaction between time and treatment group was not significant, *F*(8,242) = 0.85, *p* = 0.558. On average, patients who received GSH had higher BMI scores than those who received another treatment (see Figure 2). This pattern of results did not change after adjusting for baseline age, DUED, or waiting time for treatment, but both age (*p* < 0.001) and DUED (*p* = 0.002) predicted BMI significantly. This reflected positive associations between higher age (estimate = 0.39) and longer DUED (estimate = 0.07) and BMI. The pattern of results also remained when adding number of treatment sessions, which predicted BMI significantly (*p* < 0.001) with a negative association between higher number of sessions and lower overall BMI (estimate = −0.16) (see Table S1).

### 3.4 Discussion

This study compared use of, and outcomes from, GSH, CBT‐T, group CBT‐ED, individual CBT‐ED, and “other” individual psychological therapies for patients with a non‐underweight binge/purge ED. To our knowledge, this is the first study to compare these treatments directly. Results provide insight into how these treatments are used in routine clinical practice and how they perform in a sample of emerging adults presenting with a recent‐onset ED.

Current UK treatment guidelines recommend GSH as the first‐line intervention for BN and BED (National Institute for Health and Care Excellence 2017). Despite this, only 11% of the current sample received GSH. An additional 24% received 10‐session CBT‐T. Patients with a recent‐onset ED may be particularly well suited to programme‐led and focused treatments and so this combined rate of 35% appears low. In this study, patients who received GSH and CBT‐T had similar Global EDE‐Q and CORE‐10 anxiety/depression scores compared to patients who received longer CBT‐ED. Thus, GSH and CBT‐T were not only offered to patients with ‘mild’ ED presentations. Purging was less likely to be present in patients who received GSH but binge eating was higher in the GSH group than in other conditions, suggesting greater use with BED versus BN presentations. Interestingly, this group difference in binge eating was not significant after adjusting for covariates, and DUED was positively associated with binge eating, even in this early intervention sample.

Where GSH and CBT‐T were offered, they were similarly effective across all outcomes compared to group and individual CBT‐ED and other longer individual therapies. As this is not a non‐inferiority trial, the absence of significant difference does not allow us to conclude that these treatments were equivalent. Moreover, we recognize that patients will have been allocated to treatment conditions in non‐random ways and we are limited in our ability to understand the clinical and service‐level factors which may have influenced treatment allocations. While ED symptoms were not milder in the GSH and CBT‐T groups than in other conditions, clinicians often (and understandably) avoid offering programme‐led and focused treatments to patients with acute suicidality or deliberate self‐harm, other psychiatric risks, and high physical health risks (e.g., Dalton et al. 2024; Davey et al. 2023). Patients who received GSH and CBT‐T are therefore likely to be meaningfully different from those who received other treatments. Nevertheless, our findings are consistent with prior results showing that programme‐led and focused interventions can be effective with non‐underweight EDs (e.g., Keegan, Waller, and Wade 2022; Traviss‐Turner, West, and Hill 2017) and add to our understanding of how these treatments are used in varied ‘real world’ settings.

Treatment completion rates were comparable across conditions and were high overall (83%) in this early intervention sample. Treatment length did vary, however, and deviated from the standard protocol of 20 sessions of CBT‐ED for non‐underweight patients and 10 sessions of CBT‐T. The mean number of individual CBT‐ED sessions attended was 11, although with a large range around this (1–53 sessions). For group CBT‐ED the mean number of sessions was 8, equivalent to the mean for CBT‐T, although again with considerable variability (1–26 sessions for group CBT‐ED and 1–20 for CBT‐T). The relatively low mean session numbers may reflect early intervention patients requiring fewer sessions, on average, to achieve a good outcome than patients with longer‐standing EDs. The extension of session numbers, in a small number of cases, to more than twice the recommendations for CBT‐T and CBT‐ED also speaks to the personalization of treatment via shortening *and* lengthening of treatment length. It is not clear if treatment extensions occurred when prior sessions had not been beneficial or reflected evidence‐based but personalized practice (e.g., if a patient was experiencing benefits but needed additional time to extend progress, or to navigate life events such as starting university) (Richard‐Kassar et al. 2023). Irrespective, these findings arguably add to the case for offering non‐underweight early intervention patients GSH or CBT‐T first (unless specifically contraindicated), with longer CBT‐ED reserved for those patients who do require additional or longer‐term support. This approach is consistent with existing clinical guidelines (National Institute for Health and Care Excellence 2017) and our finding that number of treatment sessions did not, overall, predict clinical outcomes. Information on when patients were stepped up to other treatments was variably available in this sample, but in most cases, GSH and CBT‐T were offered as standalone interventions rather than being part of a stepped or augmented care pathway. This may disadvantage patients who are not able to benefit from focused interventions, while also putting clinicians off from offering these interventions if they are unsure over whether the patient will benefit.

The primary focus of this study was GSH, CBT‐T, and CBT‐ED. Nevertheless, it is interesting to reflect on the 136 patients who received a non‐CBT‐based intervention. These patients waited longer for both assessment and treatment than other patients, suggesting additional barriers or delays to engagement with the ED service right from the point of initial referral. They also started treatment with higher anxiety and depressive symptoms, and higher rates of purging, than other patients. As has been found in prior comparisons of CBT‐ED and non‐CBT‐based interventions (e.g., Miniati et al. 2018), this group took longer to experience behavioral reductions in ED symptoms than other patients, with no substantial reductions from pre‐ to mid‐treatment. They did then catch up between mid‐treatment and post‐treatment. In contrast, we observed an increase in binge eating (and to a lesser extent purging) from mid to post‐treatment for patients who received group CBT‐ED, which was significantly different to the stable or decreasing symptoms seen over this time period in other treatment groups. A prior systematic review found that group psychotherapy for EDs (including group CBT‐ED) performed comparably to individual therapy (Grenon et al. 2017), so our finding was unexpected. With the development of CBT‐T, services may now offer this in place of group CBT‐ED and/or be calibrating who is offered CBT‐ED relative to other treatments. Current UK guidelines also differ in the advice given around CBT‐ED for BED (where group treatment is recommended as part of a stepped care approach) relative to BN (where group treatment is not specifically advised). Continuing to evaluate group CBT‐ED relative to GSH, CBT‐T, and individual CBT‐ED would help improve understanding of how these treatments compare, and who they are best suited to. It is also unclear if services offered group CBT‐ED for FREED patients specifically or as a mixed group with FREED and non‐FREED patients. It is plausible that emerging adults with a recent‐onset ED would engage differently in an early intervention‐focused group relative to a group with more diverse ED presentations. This possibility could be investigated via future qualitative and quantitative research.

It is also noteworthy that 37 patients received brief psychoeducation or motivational interviewing support only. There is growing interest in the potential benefits of single session interventions (SSIs) in mental health generally and EDs specifically (e.g., Schleider, Smith, and Ahuvia 2023) and clinicians offering psychoeducation may benefit from formalized, evidence‐based protocols in how to provide this support as a standalone intervention. More work is needed in this area, including research on how single session or very brief interventions may sit alongside existing focused treatments like GSH and CBT‐T. There is some evidence that SSIs can predict subsequent response to treatment, which speaks to the potential for the ED field to move away from stepped care to more personalized augmented treatment decisions based on assessment information plus early response to treatment (Wade 2023).

We found that mean pre‐treatment BMI was higher in the GSH group than in other conditions. This may correlate with the higher rates of binge eating seen in GSH patients. It is also possible that weight bias plays a role in treatment decisions, such that patients with higher weights are more likely to be offered a programme‐led intervention than patients with lower weights. This would be consistent with prior research showing that healthcare professionals are less likely to perceive ED symptoms as severe when they're reported by larger bodied individuals, and are more likely to offer programme‐led or focused treatments to these patients (McEntee, Philip, and Phelan 2023). We also found that higher BMI was associated with receiving a lower number of treatment session, on average and across models.

The possible impact of weight bias could be investigated further as part of research into how decisions are made about treatment provision. Our findings suggest that programme‐led and focused treatments may be under‐utilized but we do not have information on factors that influenced treatment decisions. This is an important area for future research, as we know that clinical outcomes can be improved with shorter waiting times (Carter et al. 2012) and DUED (McClelland et al. 2018), but that the demand‐capacity gap undermines access to timely evidence‐based care (Kazdin, Fitzsimmons‐Craft, and Wilfley 2017). Improving our understanding of when GSH and CBT‐T are offered, and when not, and when these treatments are effective, and when not, will help to improve treatment matching and optimum use of resources (Davey et al. 2023).

This study has several advantages, including comparison of all evidence‐based CBT interventions for EDs (GSH, CBT‐T, CBT‐ED), consideration of multiple outcomes (depressive/anxiety symptoms as well as ED symptoms), and the use of a large, naturalistic, national sample. A major limitation is the amount of missing data and associated small group sizes for some analyses (particularly for CORE‐10 scores). This reflects data being collected in routine clinical practice in 54 different NHS Trusts, typically in labor‐intensive, non‐automated ways. Data of this type can be described as Flawed Uncertain Proximal and Sparse (FUPS) and are not uncommon in routine healthcare settings (Wolpert and Rutter 2018). We acknowledge that results may not generalize to other contexts, that patients will have been allocated to treatments in non‐random ways, and that replication and extension of our results are important. Improving the quality of routinely collected data is a key challenge for FREED and UK ED services more generally. A new, in‐process Eating Disorders Clinical Research Network (https://www.kcl.ac.uk/research/eating‐disorders‐clinical‐research‐network) provides hope for future improvements in data collection. Other limitations of this work include reliance on services accurately reporting the treatments they provided and a lack of corroboration over whether treatments were delivered according to evidence‐based protocols. For GSH, there was also variability in the type of GSH provided, both in relation to the source of information (book vs. online programme) and the format and number of support sessions offered. We were not in a position to conduct moderator or mediator analyses and do not have data on additional variables which may help to explain why some patients were offered focused interventions and some were not (e.g., baseline self‐reported motivation to change, comorbid diagnoses). We draw on data collected before, during, and after the COVID‐19 pandemic and so some patients may have had their treatment disrupted by the need to pause treatment and/or move treatment online. We have previously shown that FREED Network referrals increased after the pandemic, although this reflected increased referrals for AN rather than for non‐underweight presentations (Hyam et al. 2023). Others have shown that ED therapies can be effectively delivered online, with results comparable to those from face‐to‐face therapy provision (e.g., Raykos et al. 2021), but also that ED symptom severity may have increased in response to public health lockdown measures (Devoe et al. 2023). Continued evaluation of ED treatment effects post‐pandemic will help to extend our results.

Despite these limitations, this study provides new and novel data on how GSH, CBT‐T, CBT‐ED, and other ED therapies are used in routine practice and how outcomes from these treatments compare. Results suggest that GSH and CBT‐T perform comparably to longer forms of CBT‐ED, despite patients starting with similar levels of ED psychopathology and receiving fewer sessions. However, only 35% of patients were offered one of these interventions. Future studies should consider when GSH and CBT‐T are offered, and when they are not, in order to improve use of these focused interventions with patients who may benefit from them.

## Author Contributions

**Karina L. Allen:** conceptualization, formal analysis, investigation, methodology, writing – original draft. **Laura Courtney:** conceptualization, writing – review and editing. **Philippa Croft:** conceptualization, writing – review and editing. **Lucy Hyam:** conceptualization, data curation, methodology, writing – review and editing. **Regan Mills:** data curation, methodology, writing – review and editing. **Katie Richards:** data curation, methodology, writing – review and editing. **Muhammad Ahmed:** methodology, writing – review and editing. **Ulrike Schmidt:** conceptualization, funding acquisition, supervision, writing – review and editing.

## Conflicts of Interest

The authors declare no conflicts of interest.

## Supporting information


Table S1.

## Data Availability

The data that support the findings of this study are available from the corresponding author upon reasonable request.
