# Supplementary material for: Programme‐Led and Focused Interventions for Recent Onset Binge/Purge Eating Disorders: Use and Outcomes in the First Episode Rapid Early Intervention for Eating Disorders (FREED) Network
Source: Int J Eat Disord. 2024 Nov 30;58(2):389–99. doi: 10.1002/eat.24343 (PMC11861885; doi:10.1002/eat.24343)
Supplement: Supplementary file 1 — Table S1. [file EAT-58-389-s001.docx]

Supplementary Table 1

*Main effects from linear mixed models (all models include random effects of time and service)*

|  | Estimate (SE) | 95% CI | *P* |
| --- | --- | --- | --- |
| Global Eating Disorder Examination-Questionnaire (EDE-Q) scores | | | |
| Unadjusted model (n=592) |  |  |  |
| Intercept | 2.03 (0.16) | 1.72, 2.34 | <.001 |
| Time (reference = post-treatment) |  |  |  |
| Pre-treatment | 2.24 (0.11) | 2.01, 2.46 | <.001 |
| Mid-treatment | 1.13 (0.18) | 0.77, 1.49 | <.001 |
| Treatment group (reference = other individual therapy) | | | |
| GSH | -0.12 (0.18) | -0.47, 0.23 | .178 |
| CBT-T | -0.10 (0.18) | -0.39, 0.19 | .508 |
| Group CBT-ED | 0.10 (0.18) | -0.26, 0.56 | .580 |
| Individual CBT-ED | 0.04 (0.14) | -0.23, 0.31 | .771 |
| Adjusted for age, DUED, waiting time, number of treatment sessions and baseline BMI (n=498) | | | |
| Intercept | 2.26 (0.52) | 1.24, 3.29 | <.001 |
| Time (reference = post-treatment) |  |  |  |
| Pre-treatment | 2.23 (0.14) | 1.94, 2.51 | <.001 |
| Mid-treatment | 1.11 (0.28) | 0.55, 1.66 | <.001 |
| Treatment group (reference = other individual therapy) | | | |
| GSH | -0.16 (0.27) | -0.69, 0.37 | .554 |
| CBT-T | 0.35 (0.20) | -0.03, 0.74 | .074 |
| Group CBT-ED | 0.48 (0.30) | -0.10, 1.06 | .106 |
| Individual CBT-ED | 0.23 (0.18) | -0.13, 0.60 | .211 |
| Covariates |  |  |  |
| Baseline age | -0.02 (0.02) | -0.07, 0.02 | .321 |
| Baseline DUED | -0.01 (0.01) | -0.01, 0.01 | .702 |
| Waiting time | 0.01 (0.01) | 0.00, 0.01 | .084 |
| Number of sessions | -0.01 (0.01) | -0.03, 0.01 | .165 |
| Baseline BMI | -0.01 (0.01) | -0.02, 0.01 | .761 |
| Clinical Outcomes in Routine Evaluation-10 (CORE-10) scores | | | |
| Unadjusted model (n=392) |  |  |  |
| Intercept | 1.45 (0.13) | 1.19, 1.71 | <.001 |
| Time (reference = post-treatment) | | | |
| Pre-treatment | 0.78 (0.09) | 0.60, 0.95 | <.001 |
| Mid-treatment | 0.23 (0.12) | -0.01, 0.47 | .058 |
| Treatment group (reference = other individual therapy) | | | |
| GSH | -0.39 (0.14) | -0.67, -0.10 | .007 |
| CBT-T | -0.29 (0.13) | -0.54, -0.04 | .021 |
| Group CBT-ED | -0.14 (0.14) | -0.42, 0.15 | .345 |
| Individual CBT-ED | -0.18 (0.12) | -0.41, 0.04 | .110 |
| Adjusted for age, DUED, waiting time, number of treatment sessions and baseline BMI (n=302) | | | |
| Intercept | 2.18 (0.45) | 1.29, 3.06 | <.001 |
| Time (reference = post-treatment) | | | |
| Pre-treatment | 0.80 (0.12) | 0.55, 1.04 | <.001 |
| Mid-treatment | 0.14 (0.19) | -0.24, 0.51 | .469 |
| Treatment group (reference = other individual therapy) | | | |
| GSH | -0.57 (0.23) | -1.02, -0.12 | .013 |
| CBT-T | -0.12 (0.18) | -0.48, 0.24 | .518 |
| Group CBT-ED | -0.07 (0.24) | -0.54, 0.40 | .778 |
| Individual CBT-ED | -0.07 (0.17) | -0.40, 0.26 | .679 |
| *Supplementary Table 2, continued…* | | | |
|  | Estimate (SE) | 95% CI | *P* |
| Covariates |  |  |  |
| Baseline age | -0.04 (0.02) | -0.08, -0.01 | .020 |
| Baseline DUED | -0.01 (0.01) | -0.01, 0.01 | .847 |
| Waiting time | -0.01 (0.01) | -0.01, 0.00 | .088 |
| Number of sessions | -0.01 (0.01) | -0.02, 0.01 | .397 |
| Baseline BMI | 0.01 (0.01) | -0.01, 0.01 | .634 |
| Binge eating episodes per month | | | |
| Unadjusted model (n=619) | | | |
| Intercept | 0.91 (0.14) | 0.64, 1.18 | <.001 |
| Time (reference = post-treatment) |  |  |  |
| Pre-treatment | 1.40 (0.10) | 1.21, 1.60 | <.001 |
| Mid-treatment | 0.76 (0.15) | 0.47, 1.06 | <.001 |
| Treatment group (reference = other individual therapy) | | | |
| GSH | 0.34 (0.16)) | 0.01, 0.66 | .041 |
| CBT-T | -0.23 (0.14) | -0.50, 0.04 | .091 |
| Group CBT-ED | 0.37 (0.17) | 0.04, 0.70 | .028 |
| Individual CBT-ED | -0.31 (0.13) | -0.56, -0.06 | .015 |
| Adjusted for age, DUED, waiting time, number of treatment sessions and baseline BMI (n=515) | | | |
| Intercept | -0.25 (0.47) | -1.18, 0.68 | .970 |
| Time (reference = post-treatment) |  |  |  |
| Pre-treatment | 1.83 (0.14) | 1.55, 2.11 | <.001 |
| Mid-treatment | 1.20 (0.24) | 0.74, 1.66 | <.001 |
| Treatment group (reference = other individual therapy) | | | |
| GSH | 0.29 (0.26) | -0.22, 0.79 | .267 |
| CBT-T | -0.53 (0.20) | -0.92, -0.15 | .007 |
| Group CBT-ED | 0.01 (0.28) | -0.54, 0.55 | .988 |
| Individual CBT-ED | -0.51 (0.18) | -0.87, -0.15 | .006 |
| Covariates |  |  |  |
| Baseline age | -0.02 (0.02) | -0.07, 0.02 | .249 |
| Baseline DUED | 0.02 (0.01) | 0.01, 0.03 | .014 |
| Waiting time | 0.01 (0.01) | 0.01, 0.01 | .050 |
| Number of sessions | 0.01 (0.01) | -0.01, 0.30 | .123 |
| Baseline BMI | 0.04 (0.01) | 0.02, 0.05 | <.001 |
| Purging episodes per month | | | |
| Unadjusted model (n=617) |  |  |  |
| Intercept | 1.03 (0.14) | 0.76, 1.30 | <.001 |
| Time (reference = post-treatment) |  |  |  |
| Pre-treatment | 1.77 (0.10)) | 1.50, 1.97 | <.001 |
| Mid-treatment | 0.74 (0.16) | 0.44, 1.05 | <.001 |
| Treatment group (reference = other individual therapy) | | | |
| GSH | -0.94 (0.17) | -1.27, -0.60 | <.001 |
| CBT-T | -0.55 (0.14) | -0.82, -0.29 | <.001 |
| Group CBT-ED | -0.17 (0.17) | -0.50, 0.17 | .325 |
| Individual CBT-ED | -0.44 (0.13) | -0.69, -0.20 | <.001 |
| Adjusted for age, DUED, waiting time, number of treatment sessions and baseline BMI (n=514) | | | |
| Intercept | 3.42 (0.55) | 2.34, 4.50 | <.001 |
| Time (reference = post-treatment) |  |  |  |
| Pre-treatment | 2.05 (0.15) | 1.76, 2.34 | <.001 |
| Mid-treatment | 0.17 (0.27) | -0.37, 0.71 | .536 |
| *Supplementary Table 2, continued…* | | | |
|  | Estimate (SE) | 95% CI | *P* |
| Treatment group (reference = other individual therapy) | | | |
| GSH | -1.41 (0.28) | -1.97, -0.86 | <.001 |
| CBT-T | -0.49 (0.21) | -0.90, -0.08 | .019 |
| Group CBT-ED | -0.46 (0.30) | -1.04, 0.13 | .127 |
| Individual CBT-ED | -0.64 (0.19) | -1.00, -0.28 | <.001 |
| Covariates |  |  |  |
| Baseline age | -0.09 (0.02) | -0.14, -0.05 | <.001 |
| Baseline DUED | 0.01 (0.01) | -0.01, 0.02 | .251 |
| Waiting time | 0.01 (0.01) | -0.01, 0.01 | .917 |
| Number of sessions | -0.01 (0.01) | -0.02, 0.01 | .443 |
| Baseline BMI | -0.04 (0.01) | -0.05, -0.02 | <.001 |
| Body mass index (BMI) | | | |
| Unadjusted model (n=690) |  |  |  |
| Intercept | 23.80 (1.89) | 20.07, 27.53 | <.001 |
| Time (reference = post-treatment) |  |  |  |
| Pre-treatment | 1.04 (2.05) | -3.00, 5.08 | .611 |
| Mid-treatment | 2.78 (2.56) | -2.27, 7.82 | .279 |
| Treatment group (reference = other individual therapy) | | | |
| GSH | 7.35 (3.45) | 0.54, 14.16 | .035 |
| CBT-T | 3.51 (2.20) | -0.84, 7.86 | .113 |
| Group CBT-ED | 6.80 (3.78) | -0.66, 14.26 | .074 |
| Individual CBT-ED | 3.00 (2.12) | -1.21, 7.16 | .162 |
| Adjusted for age, DUED, waiting time and number of treatment sessions (n=593) | | | |
| Intercept | 15.79 (2.74) | 10.41, 21.18 | <.001 |
| Time (reference = post-treatment) |  |  |  |
| Pre-treatment | -1.52 (0.83) | -3.16, 0.12 | .070 |
| Mid-treatment | -1.33 (1.05) | -3.40, 0.75 | .209 |
| Treatment group (reference = other individual therapy) | | | |
| GSH | 5.74 (1.48) | 2.83, 8.64 | <.001 |
| CBT-T | 0.90 (1.10) | -1.26, 3.05 | .415 |
| Group CBT-ED | 2.49 (1.67) | -0.78, 5.77 | .135 |
| Individual CBT-ED | 0.03 (1.02) | -1.96, 2.03 | .975 |
| Covariates |  |  |  |
| Baseline age | 0.62 (0.12) | 0.38, 0.86 | <.001 |
| Baseline DUED | 0.08 (0.03) | 0.02, 0.14 | .009 |
| Waiting time | -0.01 (0.01) | -0.02, 0.01 | .386 |
| Number of sessions | -0.16 (0.05) | -0.25, -0.08 | <.001 |

*Note. Linear mixed models with maximum likelihood estimation were used for EDE-Q, CORE-10 and BMI scores. Generalised linear mixed models with negative binomial regression were used for binge eating and purging. For the adjusted models, baseline age, DUED and waiting time were added as covariates initially, before adding number of attended treatment sessions, and then BMI (where applicable). As the pattern of results was comparable across these adjusted models, only the final models are reported with all covariates.*

*BMI = Body Mass Index, DUED = Duration of Untreated Eating Disorder, GSH = Guided Self-Help, CBT-T = 10-session Cognitive Behavioural Therapy (CBT) for non-underweight eating disorders; CBT-ED = CBT for eating disorders*
